# Age-Dependent Protection of Insulin Secretion in Diet Induced Obese Mice

**DOI:** 10.1038/s41598-018-36289-0

**Published:** 2018-12-13

**Authors:** Elizabeth R. De Leon, Jacqueline A. Brinkman, Rachel J. Fenske, Trillian Gregg, Brian A. Schmidt, Dawn S. Sherman, Nicole E. Cummings, Darby C. Peter, Michelle E. Kimple, Dudley W. Lamming, Matthew J. Merrins

**Affiliations:** Department of Medicine, Division of Endocrinology, Diabetes, and Metabolism, University of Wisconsin-Madison, and the William S. Middleton Memorial Veterans Hospital, Madison, WI USA

## Abstract

Type 2 diabetes is an age-and-obesity associated disease driven by impairments in glucose homeostasis that ultimately result in defective insulin secretion from pancreatic β-cells. To deconvolve the effects of age and obesity in an experimental model of prediabetes, we fed young and aged mice either chow or a short-term high-fat/high-sucrose Western diet (WD) and examined how weight, glucose tolerance, and β-cell function were affected. Although WD induced a similar degree of weight gain in young and aged mice, a high degree of heterogeneity was found exclusively in aged mice. Weight gain in WD-fed aged mice was well-correlated with glucose intolerance, fasting insulin, and *in vivo* glucose-stimulated insulin secretion, relationships that were not observed in young animals. Although β-cell mass expansion in the WD-fed aged mice was only three-quarters of that observed in young mice, the islets from aged mice were resistant to the sharp WD-induced decline in *ex vivo* insulin secretion observed in young mice. Our findings demonstrate that age is associated with the protection of islet function in diet-induced obese mice, and furthermore, that WD challenge exposes variability in the resilience of the insulin secretory pathway in aged mice.

## Introduction

Diabetes is an increasingly severe problem around the globe that is estimated to affect 285 million people and predicted to increase as the populations grays^1^. With more than 25% of Americans over the age of 65 already suffering from type 2 diabetes, age is nearly unparalleled as a risk factor for the disease. This was not necessarily the case even 30 years ago, when the annual number of newly diagnosed cases remained relatively flat^2^. Age-associated risk is now comingled with obesity, a similarly potent risk factor for diabetes present in 43.5% of adult Americans^3^. While the aged have a higher prevalence of both diabetes and obesity, the relationship between these factors has only been addressed by a few studies^4–6^.

Prospective studies in mice, where obesity can be readily induced, allow the strict separation of the effects of age and obesity and have the potential to shed light on the human condition. Similarly to humans^7–11^, aged mice display insulin resistance, and maintain glucose tolerance through a combination of increased insulin levels, β-cell mass, and β-cell function^9,12–19^. While many rodent studies have addressed the impacts of obesity on insulin secretion independently (reviewed in^20–23^), there have been none addressing the impacts of age and obesity together on weight gain, glucose tolerance, and insulin secretion.

To better understand how age and obesity interact to regulate glycemic control, we investigated the physiological and metabolic impact of short-term administration of a high-fat, high-sucrose Western diet (WD) to mice from the NIA (National Institute on Aging) Aged Rodent Colony. We observed the effects of administering WD to young and aged mice for four weeks, measuring weight, glucose tolerance, β-cell mass, and glucose-stimulated insulin secretion (GSIS) *in vivo*, as well as assessing GSIS *ex vivo*. While the average effects of WD on weight and glucose homeostasis are similar for young and aged mice, we discovered a surprising degree of heterogeneity in the response of individual aged mice. Strikingly, in aged mice, but not in young mice, we discovered that WD-induced glucose intolerance and weight gain is highly correlated with the hypersecretion of insulin. This *in vivo* effect is further correlated with an age-dependent enhancement of islet function, suggesting that WD challenge exposes variability in the resilience of the insulin secretory pathway – the capacity to recover from or respond to stressors^24^ – in aged mice.

## Results

### Weight gain in aged group-housed mice, but not in young mice, correlates with Western diet-induced glucose intolerance

**Figure 1:**
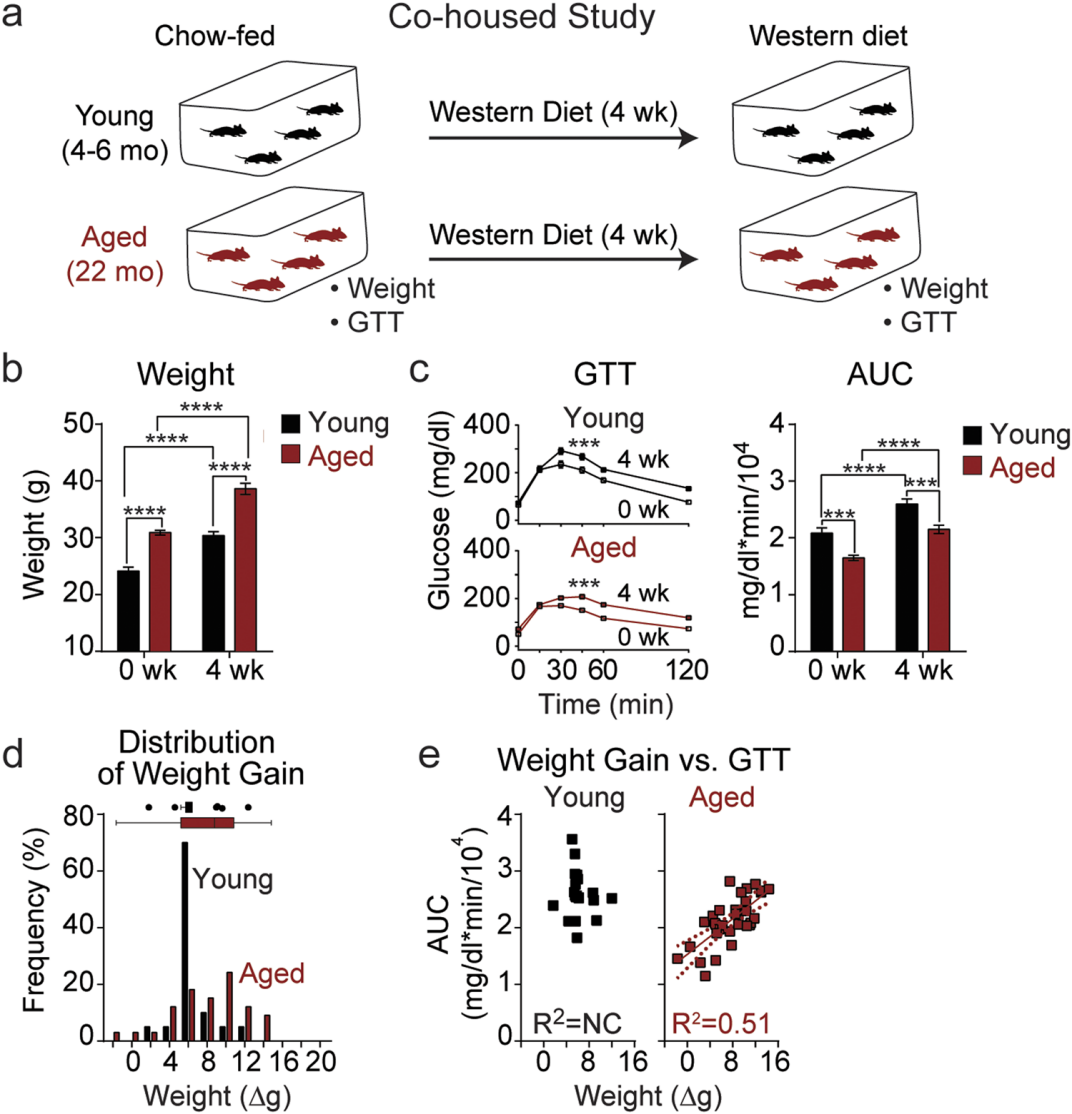
The age-dependent heterogeneity in weight gain after 4 weeks of Western diet in group-housed mice is highly correlated with impaired glucose tolerance. (**a**) Young mice (4–6 mo, black) and aged mice (22 mo, red) were co-housed in groups of 3–4 animals per cage. Mice were fed Western diet (WD) ad libitum for four weeks. Before and after diet, glucose tolerance and weight were measured. (**b**) Average weight in young (*n* = 20) and aged (*n* = 32) mice before and after WD. (**c**) Glucose tolerance test (GTT) in young (*n* = 20) and aged (*n* = 32) mice quantified by area under the curve (AUC). (**d**,**e**) Distribution of weight gain (Δg) (**d**) and correlation (**e**) between weight gain and area under the curve (AUC) in a glucose tolerance test (GTT) in young (*n* = 20) and aged (*n* = 32) mice. Data are ± SEM and were compared by paired two-way ANOVA with Sidak posttest (**b** and **c**), Tukey plot (**d**), and linear regression (**e**). NC, not correlated (P > 0.05). ***P < 0.001 and ****P < 0.0001.

We examined the susceptibility of both young (4 months of age) and aged (22 months of age) mice to weight gain and prediabetes during Western diet (WD) feeding. During this study, all mice were housed as shipped by the NIA (National Institute on Aging) Aged Rodent Colony, with 3–4 mice of the same age per cage. The weight and glucose tolerance of these mice was monitored first in chow-fed mice, and again after administration of a high-fat, high-sucrose WD for four weeks (Fig. 1a). On average, diet-induced weight gain in aged mice was very similar to that of young mice (Fig. 1b). Additionally, both young and aged mice became glucose intolerant following WD feeding, and to a similar degree (Fig. 1c). However, when considering the individual weight of each animal (as opposed to the average), we noticed that the weight gain of aged mice was highly variable (Fig. 1d). Plotting weight gain (or absolute weight, Suppl. Fig. 1) versus the area under the curve (or incremental area under the curve, Suppl. Fig. 2) during a glucose tolerance test found that weight was strongly correlated with impaired glucose tolerance (IGT) in aged mice (R^2^ = 0.51, P < 0.0001); however, there was no such correlation in young mice (R^2^ = 0.01, P = 0.61) (Fig. 1e).

### In aged mice fed a Western diet, the correlation between weight gain and glucose intolerance is independent of housing

**Figure 2:**
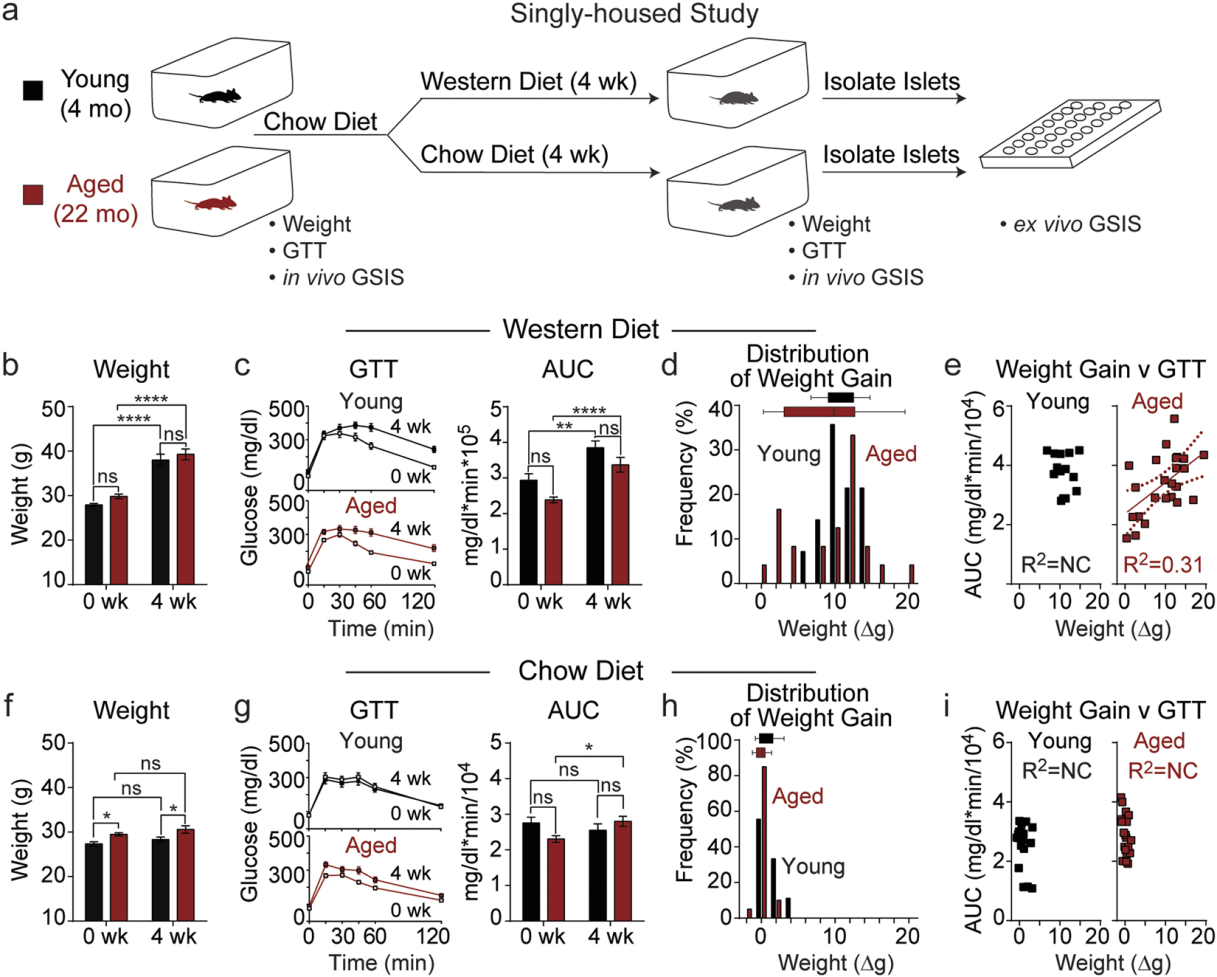
Housing-independent correlation of weight gain and impaired glucose tolerance in aged mice fed a Western Diet. (**a**) Young mice (4 mo, black) and aged mice (22 mo, red) were singly housed for a one-week adjustment period before administration of chow or Western diet (WD) ad libitum for four weeks. Before and after diet, weight, glucose tolerance, and *in vivo* glucose-stimulated insulin secretion (GSIS) weight were assessed. (**b**–**i**) Average weight (**b**,**f**), glucose tolerance (**c**,**g**), distribution of weight gain (**d**,**h**), and the correlation between weight gain and GTT AUC (**e**,**i**) were assessed before and after WD (*n* = 17–24) or chow diet (*n* = 18–22). Data are ± SEM and were compared by paired two way ANOVA with Sidak posttest (**b** and **c**), Tukey plot (**d** and **h**), and linear regression (**e**). *P < 0.05, **P < 0.01, and ****P < 0.0001.

To determine if the variability in weight gain of WD-fed aged animals was the result of co-housing induced cage dynamics, we obtained co-housed young (4 months) and aged (22 months) mice from the NIA Aged Rodent Colony, and then single-housed the animals. Following a one-week adjustment period, these mice were fed WD for four weeks as in our previous experiment. Weight and glucose tolerance were monitored before and after administration of the diet (Fig. 2a). As in the co-housed study, both young and aged mice experienced comparable average weight gain (Fig. 2b), and both young and aged mice developed IGT to a similar degree following WD feeding (Fig. 2c). Observing the frequency of individual weights before and after the diet revealed an increased distribution of weight gain following WD feeding in aged mice (Fig. 2d). Comparing glucose tolerance and weight gain in singly-housed animals again showed a strong correlation of weight gain with IGT exclusively in aged mice (R^2^ = 0.31, P = 0.035) (Fig. 2e), including when glucose tolerance was calculated from the incremental area under the curve (Suppl. Fig. 2). Thus, the correlation between weight gain and IGT is independent of housing.

To distinguish between diet and age-induced effects, and to control for any time-dependent effects of switching the mice from group to single housing, we also single-housed young and aged mice and fed them a chow diet, as opposed to a WD, for four weeks (Fig. 2a). As expected, we did not observe significant weight gain in either young or aged mice (Fig. 2f). There were no changes in glucose tolerance in young chow-fed mice during this period, but we did observe a slight increase in area under the curve in aged chow-fed mice during the four-week period (Fig. 2g). Additionally, the weight distribution of both the young and the aged mice were tightly clustered around zero or minimal weight gain (Fig. 2h), and there was no correlation between weight gain and IGT in chow-fed young or aged animals (Fig. 2i).

**Figure 3:**
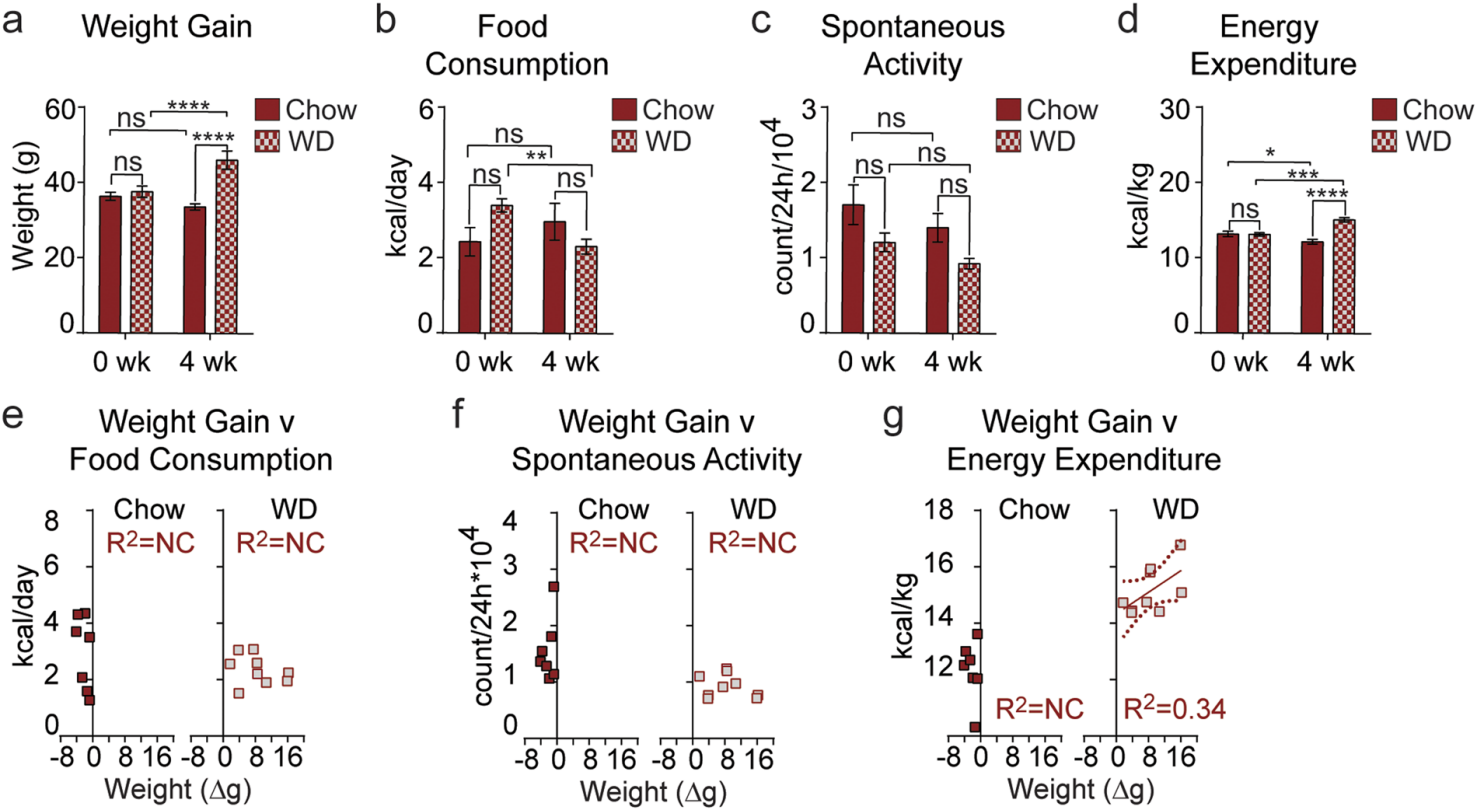
Weight gain correlates with energy expenditure in aged mice fed Western diet, but is not dependent on food consumption or spontaneous activity. (**a**–**d**) Average weight (**a**), food consumption (**b**), spontaneous activity (**c**), and average energy expenditure (**d**) in singly-housed aged mice following administration of chow or Western diet (chow, solid, *n* = 9; WD, cross-hatch, *n* = 9). (**e**–**g**) Weight gain was compared to food consumption (**e**), spontaneous activity (**f**), and energy expenditure (**g**) to acquire correlations. Data are ± SEM and were compared by paired two way ANOVA with Sidak posttest (**a**–**d**) and linear regression (**e**–**g**). *P < 0.05, **P < 0.01, ***P < 0.001, and ****P < 0.0001.

To determine if variable diet consumption was a cause of the heterogeneous weight gain of aged animals, we monitored weight gain, food consumption, spontaneous activity, and energy expenditure in an additional cohort of aged mice fed either chow or WD. As expected, average weight increased following WD-feeding, and the weight gain of individual animals was variable (Fig. 3a). Additionally, food consumption (Fig. 3b) and spontaneous activity (Fig. 3c) were not significantly changed although both trended down in aged mice fed a WD. Interestingly, energy expenditure (Fig. 3d) was increased in aged mice fed a WD and slightly decreased in aged mice on a chow diet. Comparing weight gain to these three parameters, no correlation was observed between weight gain and food consumption (Fig. 3e), or weight gain and spontaneous activity (Fig. 3f). However, a correlation (R^2^ = 0.34) was observed between weight gain and energy expenditure that was not quite significant (P = 0.100) (Fig. 3g).

### Weight gain correlates with fasting insulin and glucose-stimulated insulin secretion in aged mice

**Figure 4:**
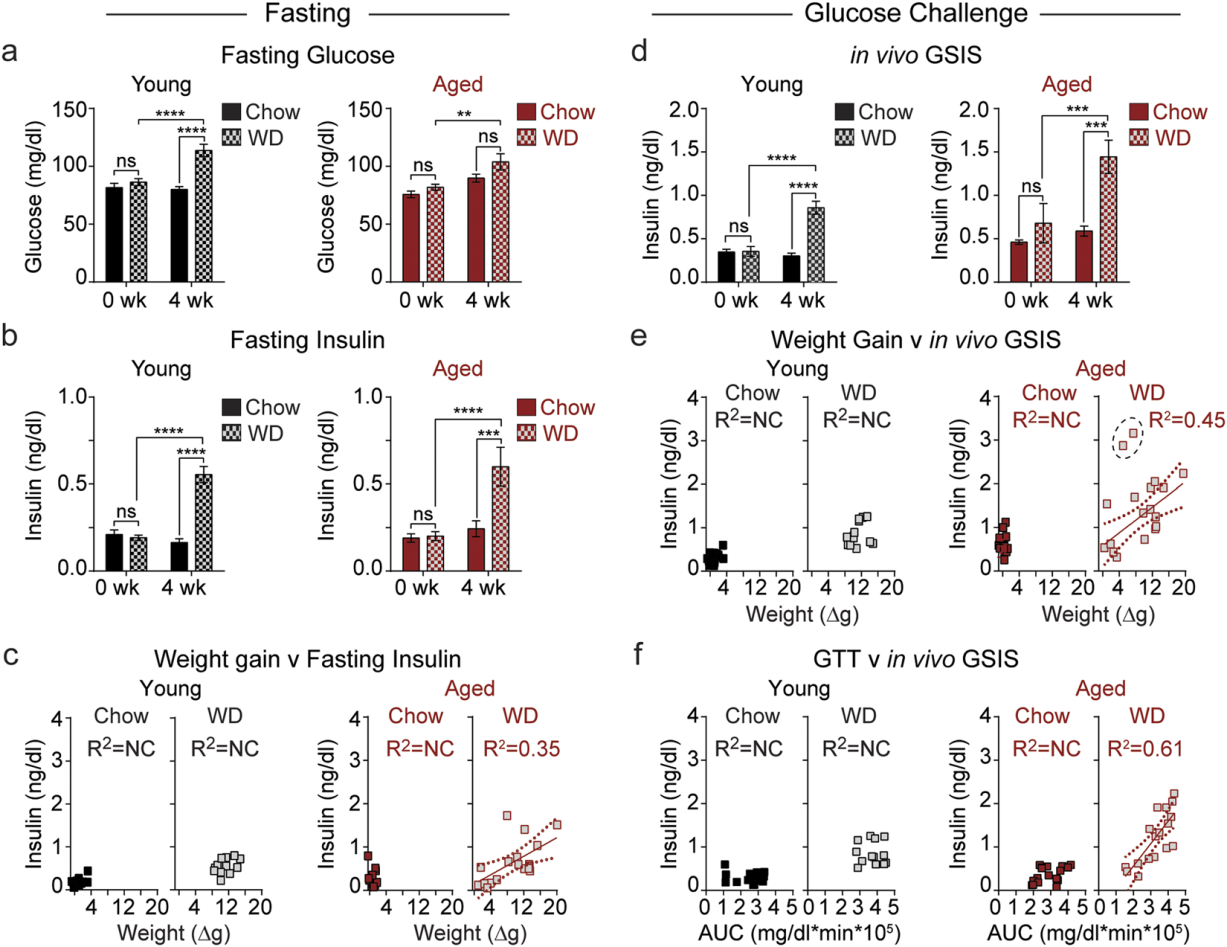
Compared with young mice, *in vivo* glucose-stimulated insulin secretion (GSIS) is enhanced in aged mice following Western diet administration. (**a**–**d**) Plasma glucose (**a**) and insulin (**b**) levels after an overnight fast in young (black) and aged (red) mice on chow (*n* = 16–17) and WD (*n* = 13–19) were plotted as a function of weight gain (**c**). Plasma insulin levels were again assessed 15 min after intraperitoneal injection of glucose (**d**). (**e**,**f**) *In vivo* GSIS plotted as a function of weight gain (**e**) and GTT AUC (**f**). The dashed circle indicates two mice excluded from regression analysis. Data are ± SEM and were compared by paired two-way ANOVA with Sidak posttest (**a**,**b**,**d**) and linear regression (**c**,**e**,**f**). **P < 0.01, ***P < 0.001, and ****P < 0.0001.

Next, to determine if the prediabetes susceptibility of young and aged mice following WD challenge differed, we monitored fasting glucose and insulin levels before and after administration of the WD (Suppl. Fig. 3). While WD-fed young mice displayed fasting hyperglycemia, aged mice maintained fasting blood glucose levels comparable to their aged-matched chow-fed counterparts (Fig. 4a). Fasting insulin was elevated to a similar degree in both young and aged mice fed a WD when compared to their age-matched chow-fed controls (Fig. 4b). Since the weight gain of the heterogeneous aged population fed a WD correlated well with IGT, we next considered the correlation of fasting insulin levels with diet-induced weight gain. We found that weight gain strongly correlated with fasting insulin levels in aged mice fed a WD (R^2^ = 0.35, p = 0.0009), but we observed no correlation in young mice (R^2^ = 0.16, p = 0.17) (Fig. 4c).

To further probe the relationship between weight gain and diabetes susceptibility in aged mice fed a WD, we proceeded to examine *in vivo* glucose-stimulated insulin secretion. Here, functional β-cell mass is assessed by monitoring levels of insulin in the plasma following glucose challenge. Both young and aged mice exhibited significant increases in average *in vivo* GSIS after consumption of a WD (Fig. 4d). Chow-fed aged and young mice did not indicate a significant increase in insulin secretion post-diet. We then tested the correlation of WD-induced weight gain with *in vivo* GSIS. While there was no correlation between weight gain and insulin secretion in young mice fed a WD (R^2^ = 0.06, P = 0.78), we observed a strong correlation between these factors in aged mice (R^2^ = 0.45, P = 0.0043) (Fig. 4e). We also asked whether *in vivo* GSIS correlates directly with IGT. We observed no correlation in young mice fed either chow or WD. In aged mice we observed a very strong correlation (R^2^ = 0.61, P = 0.0002) in the WD group that was absent in aged mice fed a chow diet (Fig. 4f). These results suggest that weight gain, glucose intolerance, and *in vivo* GSIS are strongly associated in aged mice on WD, but not in their young counterparts or aged mice fed a chow diet.

### The islets of aged mice are protected from Western diet-induced functional decline

**Figure 5:**
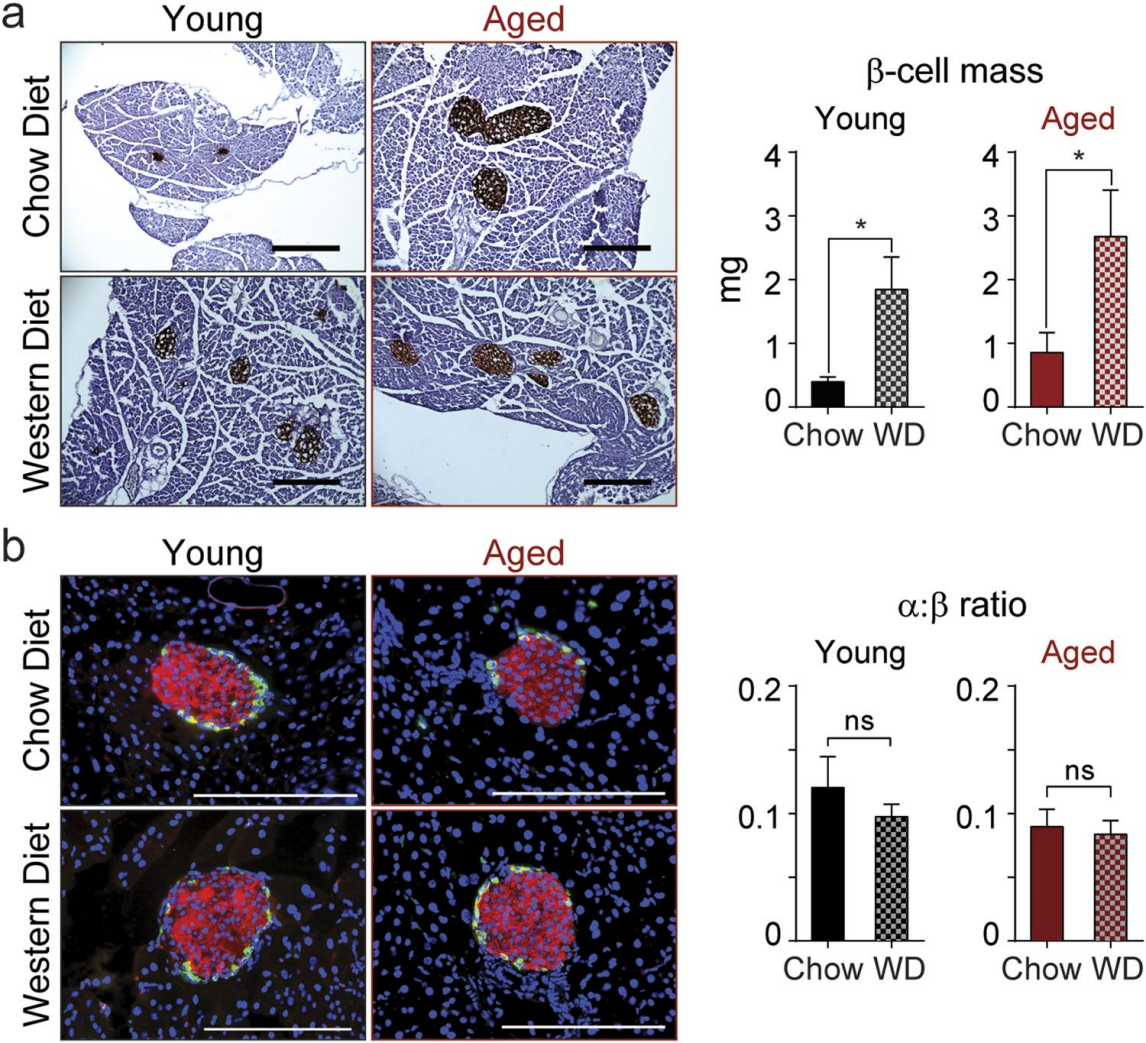
β-cell mass is increased by Western diet in both young and aged mice. Representative insulin staining and quantification of β-cell mass (**a**) and α:β-cell ratio (**b**) in chow and WD-fed young and aged mice as indicated. β-cell mass, *n* = 9 mice per group, scale bar = 400 μm; α:β-cell ratio, *n* = 3–4 mice per group, scale bar = 50 μm. Data are ± SEM and were compared by t-test. *P < 0.05.

To determine the underlying cause of increased insulin secretion in the aged mice on Western diet, we initially examined insulin-stained pancreatic sections to determine β-cell mass relative to chow-fed controls. As expected from previous work^14^, β-cell mass was slightly increased in chow-fed aged mice relative to young animals. Interestingly, aged mice exhibited a 3.1-fold expansion of β-cell mass following WD administration compared to a 4.1-fold expansion in young WD-fed controls (Fig. 5a). No changes in the α-cell: β-cell ratio were observed in either case (Fig. 5b). These findings indicate that aged mice preserve significant ability to expand β-cell mass, but also suggest that β-cell mass expansion alone cannot account for the age-dependent enhancement of insulin secretion.

**Figure 6:**
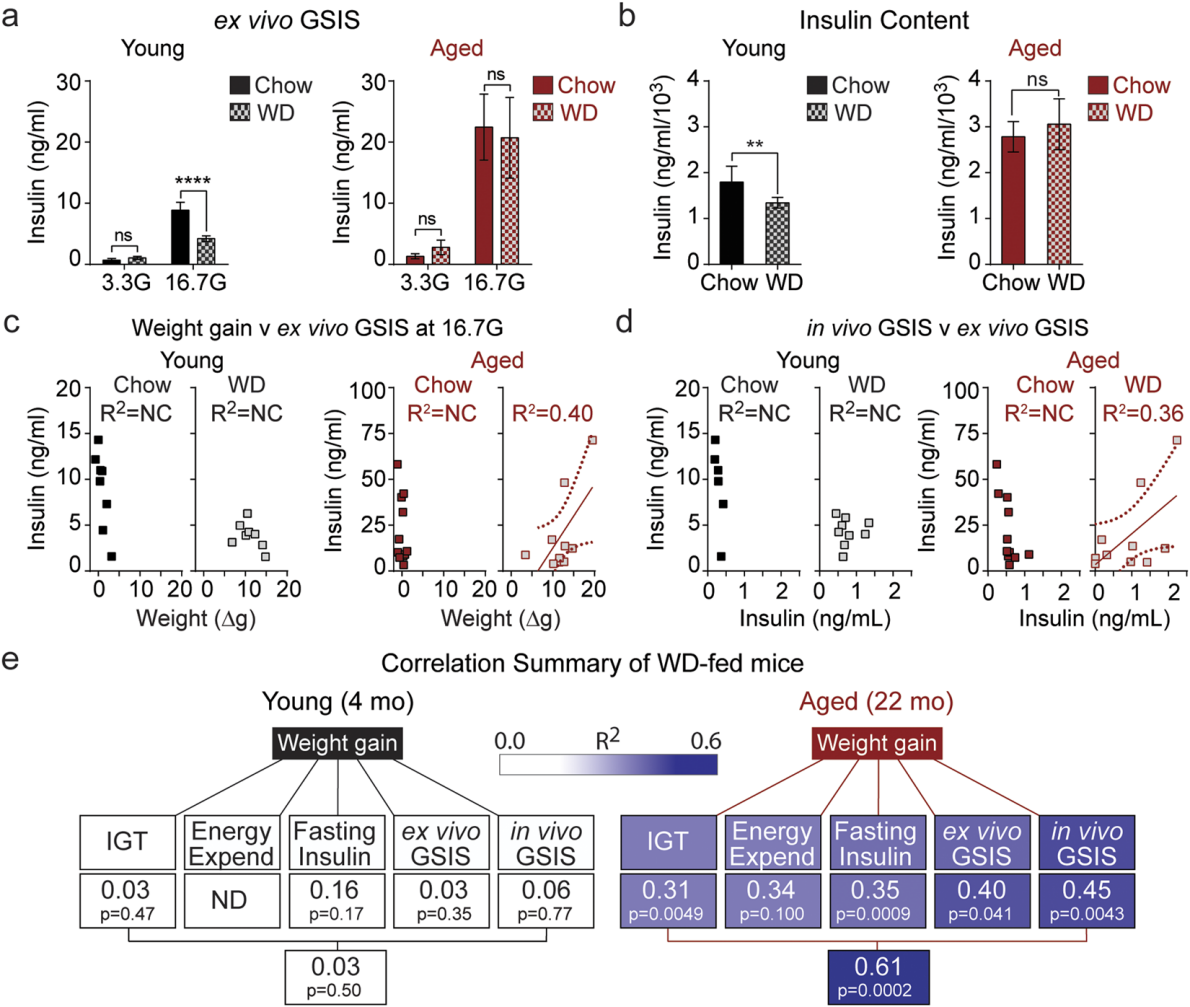
Aged mice are protected from Western diet-induced insulin secretory decline in isolated pancreatic islets. (**a**,**b**) *Ex vivo* GSIS (**a**) and insulin content (**b**) measured in pancreatic islets from young (black) and aged (red) mice on chow (*n* = 7–9) and WD (*n* = 9–11). (**c**,**d**) Correlation of *ex vivo* GSIS as a function of weight gain (**c**) and *in vivo* GSIS (**d**). (**E**) Summary of correlations (R^2^) of mice fed a Western Diet in the singly-housed study (Figs 2–6). Abbreviations: Energy Expend, energy expenditure; ND, not determined. Data are ± SEM and were compared by paired two-way ANOVA with Sidak post-test (**a** and **b**) or linear regression (**c**–**e**). **P < 0.01 and ****P < 0.0001.

We then measured *ex vivo* glucose-simulated insulin secretion. After harvesting islets from the pancreas of young and aged mice fed either chow or WD, we performed a glucose challenge and measured insulin secretion (Suppl. Fig. 3). On average, young mouse islets exhibited a significant, 53% decline in insulin secretion following WD feeding (Fig. 6a). Islets from aged mice, which were previously observed to exhibit slightly elevated *ex vivo* GSIS relative to young controls when chow-fed^17^, are protected from WD and respond normally to glucose challenge. As insulin content changes can be a strong driver of *ex vivo* GSIS, we note that islets from young WD-fed mice had reduced insulin content, while insulin content was higher in aged mice and remained unchanged following WD-feeding in the islets of aged mice (Fig. 6b). Following this analysis, we compared weight gain to *ex vivo* GSIS and observed significant correlation (R^2^ = 0.40, P = 0.041) in WD-fed mice that was not observed in the young or aged mice fed chow (Fig. 6c). Additionally, we compared *ex vivo* GSIS to *in vivo* GSIS (Fig. 6d) and determined there was a significant correlation (R^2^ = 0.36, P = 0.036) between these two parameters in aged mice on WD. These data confirm that enhanced islet function underlies the WD induced hypersecretion of insulin seen in aged mice, as well as the decline in secretory function observed in young animals. Taken together with the *in vivo* studies, these *ex vivo* analyses support a strong age-dependent association between WD-induced insulin secretion and glucose intolerance (Fig. 6e).

## Discussion

Here, we examined the effects of age on the physiological response of mice to short-term WD challenge by comparing the individual response of young and aged mice. From these studies we have uncovered three age-dependent features of diet induced obesity in mice. First is that the susceptibility to diet induced obesity in aged mice is – in stark contrast to young mice – highly heterogeneous. Secondly, the weight gain of individual aged mice but not young mice is highly correlated with a loss of glycemic control and a parallel increase in insulin secretion. Finally, insulin secretion is preserved in the islets of aged mice fed WD, but not in young mice.

While the mice used in this study were genetically identical to each other, heterogeneity resulting from WD feeding is not unheard of even in young animals^25,26^. We observed that heterogeneity with respect to weight gain and glycemic control is largely independent of housing in young mice, with group-housed or singly-housed aged mice gaining a highly variable amount of weight when challenged with a WD. Although single housing enhanced the distribution in both age groups, the variability in weight gain in aged mice continued to be much greater. Understanding the physiological and molecular basis for the greater heterogeneity of aged mice will require further research. Our initial results suggest that the variability in weight gain in aged mice is not associated with either food consumption or spontaneous activity. Another possibility is that illness may be associated with the variance in weight gain. To minimize this possibility, we discarded all data from mice with significant ulcerative dermatitis, which we have observed to be associated with morbidity and mortality^27^, as well as from mice found to have visible tumors during isolation of the pancreas. We also considered the possibility that other diseases of aging might contribute to the increased variance in weight gain we observed in aged mice. However, weight gain in aged mice fed a chow diet had a similar distribution to weight gain in young mice, making it unlikely that illness is responsible for the variance in weight gain when challenged with a WD.

Aged mice that gain little weight also display better glycemic control and are therefore protected from the effects of short-term WD feeding. Those mice that gain substantial weight are less glucose tolerant and have greater glucose-stimulated insulin secretion. Although it is tempting to assume that the weight gain leads to these phenotypes, the present experiments do not deconvolute cause and effect. The tight correlation between *in vivo* GSIS and IGT (R^2^ = 0.61, P = 0.0002) is interesting in light of the model that hyperinsulinemia drives insulin resistance and obesity^28,29^. While, classically, insulin resistance has been assumed to precede hyperinsulinemia as a result of high blood glucose^20^, our data suggest that glucose tolerance and insulin secretion heavily affect one another and that both plasma glucose and insulin are increased in the aged mice on WD which gain weight. If indeed hyperinsulinemia drives insulin resistance and obesity, then a portion of aged mice may be protected from WD-induced weight gain because the islets of these mice do not to become hypersensitive to glucose challenge.

Limitations of our study include the use of a single time point; WD studies in young animals have often used much later time points, and four weeks is early in the progression of the onset of both obesity and diabetes in mice^30^. The variability in weight gain and glycemic control may be either smaller or larger at later time points. It is also not clear if aged mouse islets are absolutely protected from WD feeding, or if they simply have improved resistance to WD such that their rate of decline is slower than young mice, which can be contrasted with mouse models of age-dependent changes in mitochondrial function^31,32^, and aged human islets where the β-cell phenotype is affected by additional genetic, epigenetic, and environmental factors^16,17,33–37^. Although these factors can lead to diabetes in some aged humans, we did not attempt to induce diabetes in our study, which modeled only the prediabetic condition (i.e. compensation, not decompensation). Other limitations are that we have not studied female mice, mice of different genetic backgrounds, or aged mice other than two years of age. While our study addresses the interaction of diet and age, by studying a single inbred strain of mice we have also not accounted for the genetic heterogeneity observed in the human population; future studies should consider exploring the effects of diet and age on weight gain and glycemic control in genetically heterogeneous populations. Notably, protection of islet function in response to a similar time course of WD administration (8 weeks) does not occur at 12 months of age in C57BL/6J mice^38^, implying that 24 month old mice are better suited for studies seeking to uncover a therapeutic route to protect aging human islets.

The results of our study have implications for best practices when studying obesity and diabetes in aged mice. Most noticeably, the increased variability of aged mice, even when genetically identical as here, suggests that studies involving aged animals will need to be larger than similar studies utilizing group-housed young animals in order to maintain adequate statistical power; e.g. using population averages to compare young and aged mice on WD, Cohen’s d suggests a sample size of 17 animals to detect a change in *in vivo* GSIS with 80% power at P < 0.05 using a two-tailed t test. We suggest that whenever possible studies utilizing diet-induced obese aged mice should be designed such that each animal serves as its own control. Further, when paired with correlation analysis, the extreme heterogeneity of aged mice could be leveraged to avoid diet itself as a confounder (i.e. by eliminating the chow fed group), particularly for islet phenotypes where insulin secretion is highly correlated with IGT (R^2^ = 0.61, P = 0.0002).

While the molecular mechanisms driving the increased variability of aged mice remains to be determined, age-dependent changes in the epigenome^16^ could be one potential explanation for the variability in the response to WD challenge; other groups have utilized diet induced obesity as a starting point to replicate and investigate epigenetics as an underlying cause of obesity, and it could be a strong next step to determine the underpinning of this heterogeneity^25,26^. Identifying the mechanisms underlying the heterogeneous response also has significant implications for aging research. We hypothesize that WD challenge reveals variability in the resilience of aged mice^24^. While it remains to be determined if longevity and frailty correlate with resilience to WD, a very short-term WD challenge of the type we employed here could be an informative and rapid way to predict healthy aging, which could be useful in the evaluation of candidate geroprotectors.

## Experimental Procedures

### Animals

All methods were carried out in accordance with their guidelines and regulations of the Institutional Animal Care and Use Committee of the William S. Middleton Memorial Veterans Hospital, who approved all animal studies required for this work. Aged (22 mo) and young (4–6 mo) C57BL/6J male mice used for this study were acquired from the National Institute on Aging (NIA) Aged Rodent Colony. In the co-housed study mice were housed as shipped from the NIA Aged Rodent Colony, and weight and glucose tolerance were monitored. The mice were then placed on Western Diet (Envigo TD.88137; kcal: 42% fat, 42.7% carbohydrate, 15.2% protein) for a period of four weeks. Weight, and glucose tolerance was again monitored. In the singly-housed studies mice were initially co-housed as shipped, and were then singly housed one week prior to the first assay. Weight, glucose tolerance, and *in vivo* GSIS assays were performed, and the mice were then placed on a Western Diet for a period of four weeks. Weight, glucose tolerance, and *in vivo* GSIS assays were again performed. Mice were subsequently euthanized and islets isolated for *ex vivo* GSIS, or intact pancreas was isolated for determination of β-cell mass as previously described^39^. Islets/pancreas were not isolated from mice found to have significant tumors following euthanasia.

### *In vivo* studies

Glucose tolerance tests were performed by fasting mice overnight for 16 hours and then injecting 1 g/kg (when GSIS was not determined) or 2 g/kg glucose (for simultaneous assessment of GSIS) intraperitoneally. Glucose measurements were collected and measured via the Bayer Contour blood glucose meter and test strips 15, 30, 45, 60, and 120 minutes post injection^40^. For GSIS, fasting insulin and *in vivo* GSIS were determined by measuring glucose and collecting tail blood via Sarstedt EDTA tubes just prior to the glucose injection and at the 15-minute time point. Insulin levels in EDTA plasma were determined with the Crystal Chem Mouse Insulin ELISA kit following the manufacturer’s protocol. We determined mouse body composition using an EchoMRI 3-in-1 Body Composition Analyzer (EchoMRI^TM^, Houston, TX, USA) according to the manufacturer’s procedures. To assay metabolic physiology (O_2_, CO_2_, food consumption) and spontaneous activity, we acclimated mice to a Columbus Instruments Oxymax/CLAMS metabolic chamber system (Columbus Instruments, Columbus, OH, USA) for approximately 24 hours prior to data collection, and data from a continuous 24 hour period was then selected for analysis as previously described^41^.

### Islet Isolation

Using 3–5 mL of 0.67 mg/mL collagenase (C7657 Sigma) and 0.2 mg/mL BSA in Hanks Buffered Salt Solution (HBSS) (Invitrogen), mouse pancreas was inflated through the common bile duct, excised, and incubated in a glass of 5 mL of Collagenase/BSA/HBSS solution for 5 min on orbital shaker at 250 rpm. At the 6^th^ minute of incubation the digest was agitated for 20 s at 375 rpm every 2 minutes until the 24^th^ minute. The pancreatic digests were washed three times by pelleting at 50 g for 2 minutes at 4 °C and washing with 30 mL of ice cold BSA/HBSS solution. Pellets were re-suspended in 1–2 mL of BSA/HBSS solution by vortex at medium speed. Islets are the hand-picked from acinar tissue in 40 mL of ice cold BSA/HBSS solution. Post isolation islets were stored in RPMI 1640 supplemented with 10% FBS (v/v), 100 units/mL penicillin, and 100 µg/mL streptomycin (Invitrogen).

### *Ex vivo* Glucose Stimulated Insulin Secretion

The *ex vivo* GSIS assay was performed in DMEM (Sigma D-5030) supplemented with 4 mM L-glutamine, 44 mM sodium bicarbonate, 10 mM HEPES and 0.2% BSA at 37 °C in 5% CO_2_. 60 islets per condition were pre-incubated for 45 min in 2 mL of DMEM containing 3.3 mM glucose. Six groups of 10 islets were then transferred to 12-well plates (Cell Treat 229112) containing 1 mL of DMEM/3.3 mM glucose per well and were incubated for 45 min. Islets were then transferred to a new 12 well plate containing 1 mL of DMEM containing 16.7 mM glucose and were incubated for 45 min. For content measurements the islets were then transferred to 500 µL cell lysis buffer containing 20 mM Tris-HCl (pH 7.5), 150 mM NaCl, and 1% Triton-X. All samples were frozen at −30 °C prior to assay by ELISA. Reagents are from Sigma unless stated otherwise.

### Insulin ELISA

ELISA was used to measure insulin secretion as a percentage of total islet insulin content. 96-well high-binding plates (Corning 3690) were coated overnight with 3 ug/mL (50 µL/well) of anti-insulin primary antibody (Fitzgerald Industries International Research 10R-I136a) diluted 1:2500 in PBS. Plates were blocked for 1 h with 100 µL/well PBS containing 4% BSA (Sigma A-7888). Following incubation plates were emptied and 25 µL/well of insulin standards (Millipore 24304391/8013-k, 0.1–10 ng/mL), secretion media, or islet lysate were added to the plate and incubated for 1 h. 25 µL/well of secondary antibody (Fitzgerald Industries International Research 10R-I136bBT) diluted 1:1000 in PBS with 1% BSA was added to each well. Plates were gently mixed and incubated for an additional hour. Plates were then emptied and washed three times with 100 µL/well of wash buffer (50 mM Tris, 0.2% Tween-20, pH 8.0). Following this 50 µL/well of 1 µg/mL of streptavidin-HRP (Pierce 21126) in PBS with 0.1% BSA was added to the plate and incubated for 30 min. Again, plates were washed three times with wash buffer. 50 µL/well of 16 µmol/mL of *o*-phenylenediamine (Sigma P-5412) dissolved in citrate buffer (0.1 M citrate-phosphate, 0.03% H_2_O_2_ at pH 5.0) was added to the plate to develop for 3–5 min. 50 µL/well of 18 mM sulfuric acid was added to quench the reaction. Following this absorbance was determined at 492 nm by plate reader (TECAN Infinite M1000 Pro). Insulin content was calculated by comparison to known standards. All products unlisted are from Sigma.

### Measurement of β-cell mass

Similar to our previous report^39^, mice were euthanized under anesthesia with CO_2_ followed by cervical dislocation. The pancreas was immediately dissected, weighed, and fixed in 10% formalin on ice for 30 minutes. Pancreata were then washed in PBS and transferred through a series of solutions, beginning with 30% sucrose in PBS, 1:1 30% sucrose:OCT, and OCT before cryopreservation in OCT and storage at −80 °C. 10-micron sections, separated by 200 microns, were cut on positively charged slides. For each pancreas, one slide, containing 3 distinct sections, was post-fixed, quenched of peroxidase activity with 3% H_2_O_2_, and immunohistochemically labeled using guinea pig anti-insulin primary antibody (Dako A056401-2), diluted 1:500 in antibody diluent, and co-stained with hematoxylin (Sigma, GHS280). Slides were imaged using an automated pan-and-stich microscope at 10× (Evos). β-cell fractional area was determined by quantifying the percent of insulin-positive pancreas area as a total of the full pancreas area for each section, followed by averaging of 3 distinct sections per mouse. Images were analyzed using ImageJ software (National Institutes of Health, Bethesda, MD) with shading correction. β-cell mass was calculated by multiplying β-cell fractional area by the pancreatic wet weight.

### Measurement of α:β-cell ratio

Three sections from each frozen pancreas were post-fixed washed with PBS and blocked with serum-free blocking agent (Dako X-0909). Anti-glucagon (1:150) (Cell Signaling, #2760) and anti-insulin primary antibodies (1:75) (Abcam, AB7842) were applied to sections for overnight treatment at 4 °C. After 1-hour treatment with FITC-anti-rabbit (1:200) (Jackson Immuno Research, 711-095-152) and Cy3-Anti-guinea pig secondary antibodies (1:400) (Jackson Immuno Research, 707-165-148), sections were counter-stained with DAPI. Slide images were captured using uniform exposure settings on a Leica DM4000B microscope (Leica Microsystems, Wetzlar, Germany) and photographed with a Retiga 4000 R digital camera (QImaging Systems, Surrey, BC, Canada). ImageJ was used to manually count glucagon and insulin-positive nuclei, respectively. The ratio of glucagon + to insulin + nuclei for each islet were calculated and averaged for each animal.

### Statistics

Data are expressed as means ± SE. Mice found to have significant tumors either prior to or following euthanasia were excluded from all analyses. Data from mice that developed severe ulcerative dermatitis or that died during the course of the experiments were likewise excluded, as were two singly-housed mice with abnormal weight gain confirmed by the Tukey outlier test. Statistical significance was determined using one- or two-way ANOVA with Sidak multiple-comparisons test post hoc or Student’s t-test as appropriate. Differences were considered to be statistically significant at P < 0.05. R^2^ values were determined by linear regression and power analysis by Cohen’s d. Statistical calculations were performed with GraphPad Prism excepting Cohen’s d, performed with R.

## Electronic supplementary material

Supplementary Figures

## Data Availability

Materials, data, and associated protocols will be made available upon request.
