# Supplementary material for: Age-Dependent Protection of Insulin Secretion in Diet Induced Obese Mice
Source: Sci Rep. 2018 Dec 13;8:17814. doi: 10.1038/s41598-018-36289-0 (PMC6292902; doi:10.1038/s41598-018-36289-0)
Supplement: Supplementary file 1 — Supplementary Figures [file 41598_2018_36289_MOESM1_ESM.docx]

**Age-Dependent Protection of Insulin Secretion in Diet Induced Obese Mice**

Elizabeth R. De Leon^1^, Jacqueline A. Brinkman^1^, Rachel J. Fenske^1^, Trillian Gregg^1^, Brian A. Schmidt^1^, Dawn S. Sherman^1^, Nicole E. Cummings^1^, Darby C. Peter^1^, Michelle E. Kimple^1^, Dudley W. Lamming^1^*, and Matthew J. Merrins^1^*


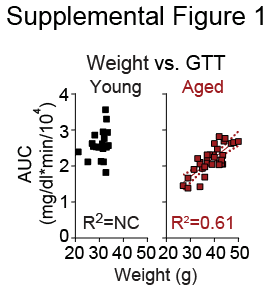


**Supplemental Figure 1. The age-dependent heterogeneity in absolute weight after 4 weeks of Western diet in group-housed mice is highly correlated with impaired glucose tolerance.**  Young mice (4-6 mo, black) and aged mice (22 mo, red) were co-housed in groups of 3-4 animals per cage. Mice were fed Western diet (WD) ad libitum for four weeks. Before and after diet, glucose tolerance and weight were measured. The graphs shows the correlation between weight and GTT AUC in WD-fed young (*n* = 20) and aged (*n* = 32) mice. Data were compared by linear regression: aged, R^2^=0.61, P<0.001; young, R^2^=0.07, P=0.25.


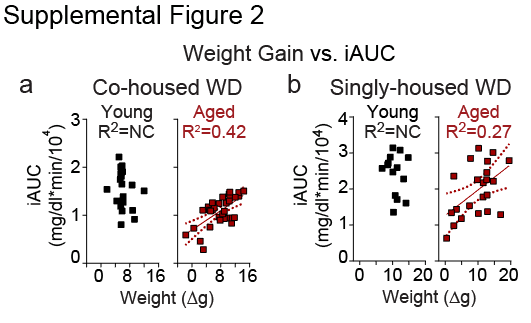


**Supplemental Figure 2. The age-dependent heterogeneity in weight gain after 4 weeks of Western diet is highly correlated with incremental AUC in a glucose tolerance test in singly-housed and co-housed mice.**  Young mice (4-6 mo, black) and aged mice (22 mo, red) were co-housed in groups of 3-4 animals per cage (**A**) or singly-housed (**B**) as in Figures 1 and 2, respectively. Mice were fed Western diet (WD) ad libitum for four weeks. Weight gain and incremental area under the curve (iAUC) data were compared by linear regression. In the co-housed animals, the correlation between weight gain and iAUC is highly significant in aged mice (R^2^=0.42, P<0.0001), but not young mice (R^2^=0.06, P=0.30). In singly-house mice, the correlation between weight gain and incremental AUC (iAUC) is significant in aged mice (R^2^=0.27, P=0.035), but not young mice (R^2^=0.03, P=0.53).


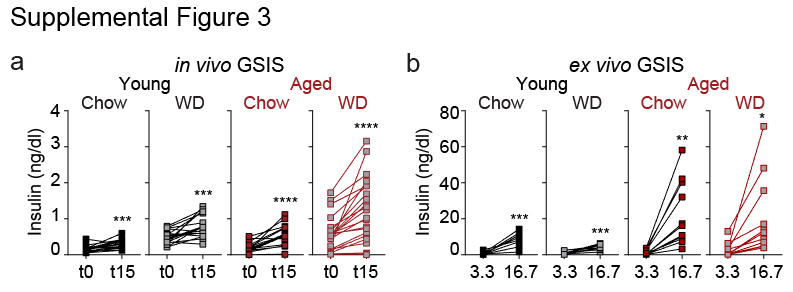


**Supplemental Figure 3. Compared with young mice, *in vivo* and *ex vivo* glucose-stimulated insulin secretion (GSIS) is enhanced in aged mice following Western diet administration.** (**A**) Plasma insulin levels before and after an overnight fast in young (black) and aged (red) mice on chow (*n* = 16-17) and WD (*n* = 13-19). Data is redisplayed from Figures 4a and 4d. (**B**) *Ex vivo* GSIS (A) and insulin content (B) measured in pancreatic islets from young (black) and aged (red) mice on chow (*n*= 7-9) and WD (*n* = 9-11). Data is redisplayed from Figure 6a. All data were compared by paired t-test. *P < 0.05, **P < 0.01, ***P < 0.001, and ****P < 0.0001.
